# Male Strategic Association With Mating Partners Under Varying Social Contexts in a Livebearing Fish

**DOI:** 10.1002/ece3.73175

**Published:** 2026-02-26

**Authors:** Arezo Shamsgovara, Lennart Winkler, Alvin Sellin, David Wheatcroft, Niclas Kolm, John L. Fitzpatrick

**Affiliations:** ^1^ Department of Zoology Stockholm University Stockholm Sweden

**Keywords:** male mate choice, male preference, mate‐choice copying, sexual selection, sperm competition

## Abstract

Group living species are constantly facing decisions about which conspecifics to associate with. These decisions are likely guided by the benefits and costs of associations. Associating in larger groups can minimize predation risk, while also providing individuals with beneficial social information from conspecifics. By contrast, associating with multiple individuals could also increase potentially costly conflicts over resources and/or mates. Here, we examine male association strategies in a shoaling fish, the pygmy halfbeak ( *Dermogenys collettei* ), by confronting males with four different social scenarios. We found that males preferentially associated with an opposite sex pair (i.e., a female and a male) over rival males, but showed no preference when choosing between a pair and females. By contrast, the number of presented fish (one or two) did not influence male association preferences, indicating that the observed male behavior was not driven by shoaling behavior. Finally, male association preference correlated with the duration of courtship behavior that the male observed, but only under specific social scenarios. Overall, our data show that males followed informed association strategies that are primarily driven by mating opportunities. Using a simplified social environment, we illuminate which basic rules might drive association behavior in complex social groups.

## Graphical abstract

We found that in halfbeaks, a shoaling fish species, males preferentially associated with an opposite sex pair (i.e., a female and a male) over rival males, but showed no preference when choosing between a pair and females. Overall, our data show that males followed informed association strategies that are primarily driven by mating opportunities. Using a simplified social environment, we illuminate which basic rules might drive association behavior in complex social groups.

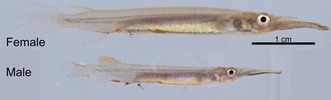

## 1 Introduction

Group living can have both benefits and costs for individual group members. Larger groups may enhance anti‐predator mechanisms through risk dilution and improved early detection of threats (Chivers and Ferrari 2015). Additionally, group living facilitates social learning, allowing individuals to acquire information by observing others' behavior and preferences (Bandura et al. 1961; Danchin et al. 2004; Smith 2021; Cirino et al. 2023). However, living in larger groups also increases the potential for competition for resources and reproductive opportunities (Clutton‐Brock and Huchard 2013). In particular, interactions among individuals in mixed‐sex groups can influence mate selection, competition, and access to breeding opportunities (Reichard et al. 2004; Aronsen et al. 2013; Winkler et al. 2023). Ultimately, the trade‐offs associated with social living are shaped by group size, ecological conditions, and the interplay between cooperation and competition among individuals (Brown 1982; Creel and Creel 1995; Chivers and Ferrari 2015).

Mating decisions can be influenced by the social environment in a group, which plays a crucial role in shaping reproductive dynamics (Reichard et al. 2004; Aronsen et al. 2013; Winkler et al. 2023). While mate choice has been extensively studied in females, reflecting traditional assumptions that males are the less choosy sex (Bateman 1948; Westneat et al. 2000; Kokko and Johnstone 2002; Bierbach et al. 2011), research also indicates that males exhibit mating preferences (Bonduriansky 2001; Sæther et al. 2001; Wong et al. 2004; Plath, Seggel, et al. 2006; Plath et al. 2009; Bierbach et al. 2011). Males may perform mate choice when sperm production is costly, when they provide parental care (Milinski and Bakker 1997; Wong and Jennions 2003; Kokko and Jennions 2008; Bierbach et al. 2011), or in social groups where only a small proportion of females are receptive at a given time (A. E. Magurran 2005; Bierbach et al. 2011). For males to be able to perform adaptive mate choice, there are three main pre‐requisites (Bonduriansky 2001; Kokko and Johnstone 2002; Edward and Chapman 2012): First, perceivable variation in female quality. Second, that males cannot mate with every available female. Third, the benefits of choosing outweigh the costs of assessing female quality. Hence, in complex social groups male association strategies should maximize the number and quality of potential partners, all the while avoiding rivals to minimize costly male–male combat and sperm competition (Kokko and Jennions 2008; Klug et al. 2010; Venner et al. 2010; Bierbach et al. 2011).

Selecting an optimal mating partner incurs time and energy costs. To reduce these costs, individuals in group living species may rely on information gathered from mating interactions between conspecifics, which leads to non‐independent mate‐choice (Valone 1989; Westneat et al. 2000; Danchin et al. 2004). The mating decisions of conspecifics can therefore lead to an increased perceived attractiveness of a potential mate (Pruett‐Jones 1992; Vakirtzis 2011; Scauzillo and Ferkin 2019; Davies et al. 2020) and may be particularly advantageous for inexperienced individuals or in situations where individual assessment of mate quality is difficult (Sirot 2001; Munger et al. 2004). Yet while copying another male's mate choice decisions can reduce the energetic and opportunity costs associated with mate searching (Schlupp and Ryan 1997; A. E. Magurran 2005; Bierbach et al. 2011), taking advantage of this social information also increases the risk of sperm competition. Males are consequently expected to balance the costs and benefits of relying on social information when making mate choice decisions (Bierbach et al. 2011; Plath and Bierbach 2011; Nöbel and Witte 2013). However, surprisingly few studies examine how variation in both male rivals and female potential mates influences male mate choice decisions (MacLaren and Rowland 2006; Sato et al. 2014; Wacker et al. 2016; McNeil et al. 2021).

Here, we test whether male association preferences change depending on social conditions using a dichotomous choice assay. Specifically, we investigated how males associate with conspecifics across four social contexts in the pygmy halfbeak ( *Dermogenys collettei* ), a shoaling, livebearing fish with documented male mate‐choice (Ogden and Fitzpatrick 2019; Ogden et al. 2020). We manipulated both the sex (i.e., female or male) and the number (i.e., one or two individuals) of stimulus fish presented to a focal male, measuring association preference compared against a male–female pair (Figure 2). In this way we manipulated the potential for male–male competition and mating opportunities presented to the focal male, while a male–female pair was used as a baseline providing both a mating opportunity (i.e., the paired, stimulus female) as well as a competitor (i.e., the paired, stimulus male). These conditions allow us to assess whether the focal male is sensitive to the number of conspecifics and preferentially associates with a pair over a single individual. Specifically, we predict that the focal male preferentially associates with two over one fish as a result of shoaling behavior. In addition, we predict that males prefer to associate with an un‐paired female over a paired female, which would indicate that males follow association strategies to minimize (sperm) competition. Furthermore, we examined whether males adjust their association preferences based on the number of females, which would indicate that social decisions are guided by increased mating opportunities. We also assessed whether males avoid male–male competition (Plath, Meyer‐Lucht, and Poschadel 2006) by adjusting their association preferences based on the number or size of rival males. We predict that focal males will avoid association with males and that this effect will increase with the number of males as well as their size. Finally, we examined if males mate preferences are sensitive to the amount of courtship behaviors that rival males direct toward females.

## 2 Materials and Methods

### 2.1 Study System

**FIGURE 1:**
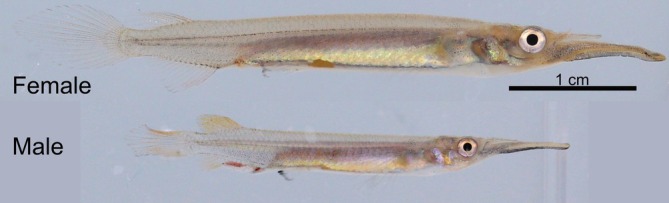
Female (upper) and male (lower) halfbeaks ( *Dermogenys collettei* ) with 1 cm scale bar. Female and male separately photographed as described below (see Section 2.6).

Pygmy halfbeaks are internally fertilizing, livebearing freshwater fish from Southeast Asia that live in mixed‐sex shoals of 6 to 128 individuals (Greven 2010; Ho et al. 2015; Devigili et al. 2021; McNeil et al. 2021). Adult pygmy halfbeaks exhibit both sexual dimorphism and sexual dichromatism (Figure 1). Females grow larger than males, while males display more vibrant red and yellow hues on their modified anal fins (Greven 2010; Devigili et al. 2021). In our experiment, females were on average 3.09 cm long (SD [cm] = 0.36; *n* = 156) and males were on average 2.38 cm long (SD [cm] = 0.11; *n* = 252; standard length excluding the beak). Female mate choice has been demonstrated to be influenced by the intensity of red colouration in males (Reuland et al. 2020; McNeil et al. 2021). In contrast, males perform distinct courtship displays by swimming underneath females and assessing potential mates based on the size of an orange‐colored ornament on the female's abdomen, known as the gravid spot, which fluctuates in size based on the reproductive stage and influences male mate choice (Ogden and Fitzpatrick 2019; Ogden et al. 2020; Daupagne et al. 2025). Additionally, agonistic interactions are common in both sexes, though males are usually more aggressive (Greven 2010; Ho et al. 2015; Devigili et al. 2021).

### 2.2 Study Population

Pygmy halfbeaks used in this study were sexually mature, wild‐caught (Johor River, Malaysia) individuals that were kept in the aquarium facility at Stockholm University, Sweden. Experimental fish were maintained in mixed‐sex groups (each with ~20 fish at roughly 1:1 sex ratio) in 80‐L stock tanks under a 12:12 h dark/light regime, at an average water temperature of 23°C. The tanks were set up with a ~2 cm layer of gravel, artificial plants, and continuous oxygenation. Their diet consisted of a mix of flake food (JBL Flakes, Neuhofen, Germany) and freshly hatched *Artemia nauplii*, provided alternating six times per week. The diet was supplemented with previously frozen, then thawed *Drosophila* every 2 to 3 days.

### 2.3 Experimental Setup

**FIGURE 2:**
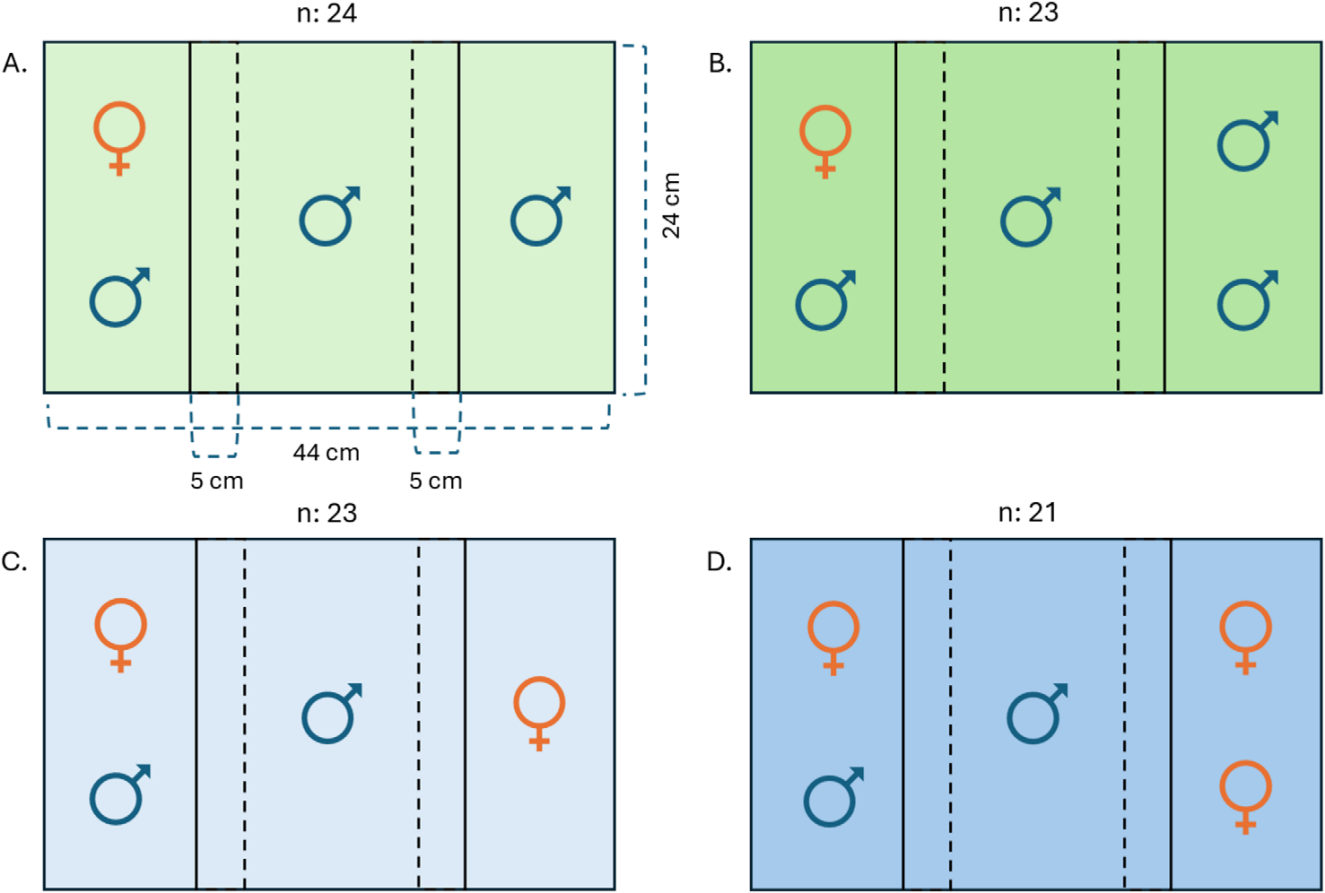
Scheme illustrating the four different experimental treatments varying the number (i.e., one (A, C) or two (B, D)) and sex (i.e., female (C, D) or male (A, B)) of presented stimuli individuals from a top view. Please note that the side at which the treatment (here right) or the pair (here left) were presented was randomized throughout the experiment. In addition, final samples sizes (*n*) are given for each treatment, as well as the tank and association zone dimensions in panel (A).

A dichotomous choice assay was used to assess male preference. The testing environment (44 cm long, 25 cm wide and 20 cm high; no substrate) consisted of a main compartment (24 cm long) which housed the focal male, and two smaller compartments (10 cm long) housing the stimuli fish (Figure 2). Each compartment had a water level of ~8 cm, allowing for male courtship behavior to occur. The compartments were separated by transparent acrylic barriers to allow visual cues, while ~2 mm perforations in the barriers facilitated the transmission of olfactory cues. Trials were recorded using a mounted camera (Grasshopper3 GS3‐U3‐41C6C, Teledyne Vision Solutions, Thousand Oaks, USA) positioned ~120 cm above the tanks and the recoding program “FlyCapture2” (Version 2.23.3.2) using 20 frames per second. Dividers were placed at a ~6° inward angle to minimize blind spots in the recordings. Association zones were defined as a 5 cm long area adjacent to the barriers. To prevent external visual distractions from the outside environment, the tanks were surrounded by opaque dividers. Four tanks with randomized treatments were set up and recorded in parallel, with one camera per two tanks.

### 2.4 Dichotomous Choice Experiment

Females were size‐matched by eye based on body size and gravid spot size for each trial to minimize size‐related differences. Indeed, female body size (*r* = 0.82, *t* value = 9.45, df = 42, *p*‐value < 0.001) and gravid spot size (*r* = 0.73, *t* value = 7.01, df = 42, *p*‐value < 0.001) correlated significantly (Pearson correlation coefficient) between the females of the same trial when measured after the experiment. For logistical reasons, males were not size matched, but all males were measured after the experiment and male size did not influence the observed association patterns (Table A5, Figure A5). The focal males and stimuli fish were sourced from separate stock tanks to prevent potential effects of familiarity. While all focal males and all females were only used once, some stimulus (or “treatment”) males were re‐used for logistical reasons (in completely novel combinations), with a minimum of 48 h of rest between experiments. The placement of stimuli was randomized and counterbalanced across trials to mitigate side bias effects. A 30‐min acclimation period was given, during which visual interactions between the focal male and stimulus fish were blocked by opaque acrylic dividers. This barrier likely also reduced olfactory cues, but we do not know to what extent. Following this acclimation period, the opaque divider was gently removed by hand, allowing the focal male to perceive visual cues from both stimuli chambers. Each trial lasted 45 min.

### 2.5 Treatments

We set up four different treatments testing the preference of a focal male for the presented stimuli. One of the stimuli was always a pair (i.e., one female and one male), which we refer to as a “pair” from here on. By contrast, the stimuli presented in the other compartment varied and we will from here on refer to them as the “treatment” group. The treatment group varied in the number (i.e., one or two individuals) and sex (i.e., male or female) of the presented fish (Figure 2). Each treatment was replicated 24 times using different focal males (*n* = 96), but final sample sizes varied slightly due to fish that escaped to different compartments of the tank (see Figure 2 for final sample sizes per treatment).

### 2.6 Measurements

The recorded footage was analyzed using “EthoVision XT” (Version 17.5.1718; Noldus 2023) to track the focal individual and quantify the duration it spent in each association zone. All focal males visited at least one of the association zones, with 87 out of 91 males visiting each zone at least once. The mean time spent in the association zones was 31 min, ranging from 8 to 44 min. Association time was used as a proxy for mate choice (Walling et al. 2010; Dougherty 2020), although it might also indicate general social behavior or shoaling behavior (especially since not all presented stimuli were potential mates). Subsequently, we used association time to calculate the strength of preference (SOP) for focal males. SOP was calculated as the time spent in the pair association zone minus the time spent in the treatment association zone, divided by the total time spent in the association zones. Hence, values are bound between 1 and −1, with 1 representing perfect association with the pair and −1 perfect association with the treatment group.

In addition, we quantified courtship behavior in the pair using the “Behavioural Observation Research Interactive Software” (BORIS; Version 8.27.10; Friard and Gamba 2016). Specifically, we counted the duration of courtship displays performed by the pair male. Courtship was defined as males assessing female receptivity by swimming closely underneath them, using the gravid spot as their observable mate selection cue (Ogden et al. 2020).

We photographed all fish laterally using a Canon EOS 800D digital camera (Canon Europa N.V., Amstelveen, The Netherlands) with a Canon EFS 60 mm objective (Canon Europa N.V., Amstelveen, The Netherlands) for morphological measures. The length was determined by measuring a segment starting at the edge of the most distal/anterior point of the eye extended to the end of the fish's body at the caudal peduncle (Reuland et al. 2020). In addition, all females were photographed from the ventral side in order to measure gravid spot size (Ogden et al. 2020). The total gravid spot area was determined by tracing around the visible orange marking on the female's abdomen. We performed all morphological measurements using “ImageJ” (Version 1.54 k; Schneider et al. 2012).

### 2.7 Statistical Methods

First, we tested if the SOP differed significantly from zero (i.e., no preference) using linear models for each of the four treatments individually. We then tested if the SOP differed between treatments using a linear model that included both the treatment sex (i.e., male or female) and the number of fish in the treatment group (i.e., one or two) as well as their interaction. We removed the non‐significant interaction in this model, as this did not significantly affect the model fit (tested via ANOVA; see Table 2). Finally, we performed post hoc comparisons between all four treatments testing for differences in SOP.

Second, we analyzed the courtship behavior of the pair males in the stimuli chambers and its relationship to the focal male's SOP. If focal males reacted to the mating decisions of stimulus males, we expected to find a stronger SOP toward the pair in trials where more courtship behavior was performed in the pair. We first analyzed the full data set, including pairs where the male did not perform any courtship behavior. Nevertheless, the focal male might only react to pairs that actually performed courtship, particularly as lack of courtship could be driven by male mating status (i.e., spent males) or stress level, as opposed to being informative of the female's quality. Therefore, in an additional analysis, we excluded trials where the pair male did not perform any courtship behavior (*n* = 25). Further, we excluded very short courtship behaviors from the analysis using a data driven threshold of 2 s, removing the lower quartile of courtship behaviors (*n* = 258 of 1128 behaviors), as such fleeting courtship behaviors are less likely to be observed by the focal male. This led to the exclusion of a further 5 trials where the focal only performed short courtships. After removing these data, 15, 17, 15, and 14 replicates remained for the one male, two male, one female and two female treatments, respectively. We log_10_ transformed courtship duration throughout improving the fit of the data to a normal distribution (estimated by visual inspection of model residuals). We calculated Spearman correlation coefficients as this test is robust at small sample sizes (De Winter et al. 2016).

Third, we explored if the general activity (i.e., distance moved as measured by the “EthoVision XT” tracking) by the focal male differed between treatments. This was done to evaluate if the treatment affected how much the focal male moved independent of any preferences, indicating levels of general exploratory behavior. Due to their ecology, halfbeaks mainly move on the surface of the water (Devigili et al. 2021), which allowed us to interpret any observed movements as reflecting horizontal movements in a 2D environment. We note that this measure does not distinguish between different spatial patterns of movement (e.g., staying near the stimulus vs. switching sides). We tested if the distance moved differed between treatments using a linear model that included both the treatment sex (i.e., male or female) and the number of fish in the treatment group (i.e., one or two) as well as their interaction. We removed the non‐significant interaction in this model, as this did not significantly affect the model fit (tested via ANOVA; see Table 2). Finally, we performed post hoc comparisons between all four treatments testing for differences in distance moved.

Fourth, we tested how the morphology of the stimulus fish (both pair and treatment individuals) related to the SOP of the focal male. Morphological characteristics, including body size or gravid spot area of females, may provide important cues for mate choice (Ogden et al. 2020) or male–male competition (Goncalves et al. 2025). We correlated the body length of all stimulus females (pair and treatment) with the SOP using linear models and Spearman correlation coefficients, using the mean body length if two females were present. Likewise, we correlated the gravid spot area (known to influence male mating behavior; H. J. P. Ogden et al. 2020) of the pair female, the mean gravid spot area of the treatment females, and the gravid spot area of the pair female relative to the mean gravid spot area of the treatment females to the SOP. Finally, we also correlated the mean body length of the treatment males and body length of the pair male relative to the mean body length of the treatment males with the SOP.

Fifth, we tested potential traits that might covary with the courtship behavior of the pair male. We correlated the duration of courtship performed by the pair male with the length of the pair female, the gravid spot area of the pair female and the gravid spot area of the pair female relative to the length of the pair female using linear models and Spearman correlation coefficients. In addition, we also tested if the courtship duration varied with treatment (i.e., the fish present on the other side of the arena). Note that in these analyses we included all courtship durations (i.e., zeroes and short courtships) in contrast to the previous analyses, as we were interested to explore the factors influencing the courtship behavior of the pair male independent of the focal male.

All analyses were performed in R (V4.4.1; R Core Team 2024) and all model fits were visually and statistically inspected using the “DHARMa” package (V0.4.7; Hartig 2024).

### 2.8 Ethical Note

Throughout the experiment we followed the ASAB/ABS Guidelines for the ethical treatment of nonhuman animals in research. Specifically, we kept handling to a minimum and allowed appropriate acclimatization times to reduce stress. We provided enrichment through plastic plants and gravel throughout, except during the behavioral measurements, so as not to interfere with the recording. After the experiments all individuals were returned to their stock tanks and no animals died during the study. We used a total of 406 sexually mature adult halfbeaks (168 females and 238 males) of at least 1 year of age. Ethical approval for all experimental procedures was obtained from the Stockholm Animal Research Ethical Board (permit numbers Dnr 3967‐2020 and Dnr 16124‐2023).

## 3 Results

### 3.1 Strength of Preference and Activity Patterns of Focal Males

**FIGURE 3:**
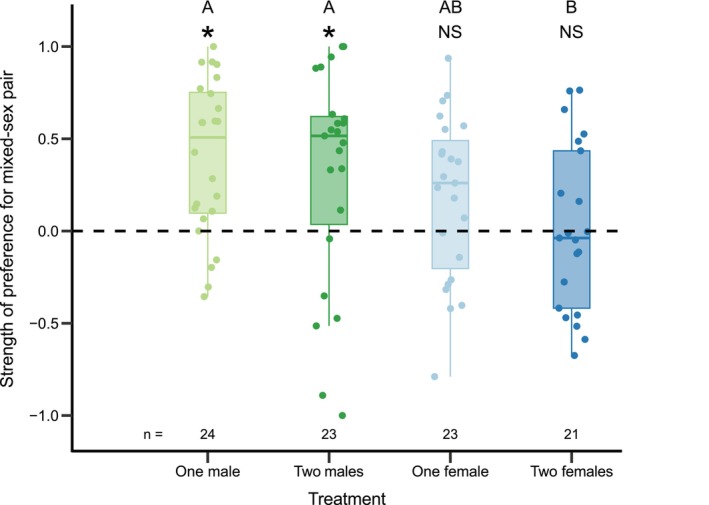
Strength of preference (SOP) of focal males toward a mixed‐sex pair, in contrast to one of four treatment groups (i.e., one male, two males, one female or two females). Green and blue colors indicate male and female treatment, respectively. Light and dark colors indicate one or two individuals in the treatment group, respectively. No preference at zero (dashed line), full preference for mixed‐sex pair at one and full preference for treatment group at minus one. Sample sizes (*n*), significant difference to zero (i.e., no preference; Table 1) indicated by *. Different letters denote significant differences between treatments (Table 2a). Boxplots show median, first/third quantiles and whiskers indicating data range excluding outliers.

First, we tested the association preferences of focal males for each of the four different treatments (i.e., one male, two males, one female or two females) compared to the pair. Focal males associated more with the pair over both the single and the pair of males (Table 1, Figure 3). Further, we found weak non‐significant evidence suggesting that focal males spend more time with the pair compared to the single female, but focal males did not associate more with a pair compared to two females (Table 1, Figure 3). Overall, the sex of the treatment group, but not the number of fish in the treatment group, significantly predicted the SOP of the focal male (Table 2a). Post hoc tests revealed that SOP was significantly higher in the treatment with one and two males compared to the treatment with two females (Table 2a, Figure 3). There were no other significant differences in SOP between the treatment groups (Table 2a, Figure 3).

**TABLE 1:** Strength of preference (SOP) for the pair in contrast to four different treatments (i.e., one male, two males, one female or two females).

Treatment	Mean SOP (SE)	df	*t* value	*p*
One male	0.39 (0.09)	23	4.57	**< 0.001**
Two males	0.31 (0.12)	22	2.54	**0.019**
One female	0.18 (0.09)	22	1.95	0.064
Two females	0.01 (0.10)	20	0.12	0.902

*Note:* Significant *p*‐values in bold.

**TABLE 2:** Comparisons of (a) strength of preference (SOP) and (b) distance moved between the treatment sex (i.e., male or female) and the number of fish in the treatment group (i.e., one or two), as well as post hoc comparisons between all treatment groups.

Treatment	Estimate	SE	*t* value	*p*
(a) Strength of preference based on sex and number of fish^a^				
Sex (male)	0.25	0.10	2.53	**0.013**
Number of fish (two)	−0.12	0.10	−1.23	0.222
Post hoc comparisons				
One male—two males	−0.08	0.14	−0.59	0.558
One female—two females	−0.17	0.14	−1.15	0.252
One male—one female	−0.21	0.14	−1.52	0.132
One male—two females	−0.38	0.14	−2.65	**0.010**
Two males—one female	−0.13	0.14	−0.92	0.359
Two males—two females	−0.30	0.14	−2.05	**0.043**
(b) Distance moved based on sex and the number of fish^b^				
Sex (male)	−608.20	186.10	−3.27	**0.001**
Number of fish (two)	84.06	186.10	0.45	0.653
Post hoc comparisons				
One male—two males	36.84	260.23	0.14	0.888
One female—two females	134.60	269.20	0.50	0.618
One male—one female	561.00	260.20	2.16	**0.034**
One male—two females	695.60	266.50	2.61	**0.011**
Two males—one female	524.10	263.00	1.99	**0.049**
Two males—two females	658.70	269.20	2.45	**0.016**

*Note:* dfs were 88 and 87, respectively. Reference group given in brackets. Significant *p*‐values in bold.

^a^ Interaction removed, as it did not significantly improve the model fit (*F* = 0.17, df = 87, *p* = 0.675).

^b^ Interaction removed, as it did not significantly improve the model fit (*F* = 0.07, df = 86, *p* = 0.795).

**FIGURE 4:**
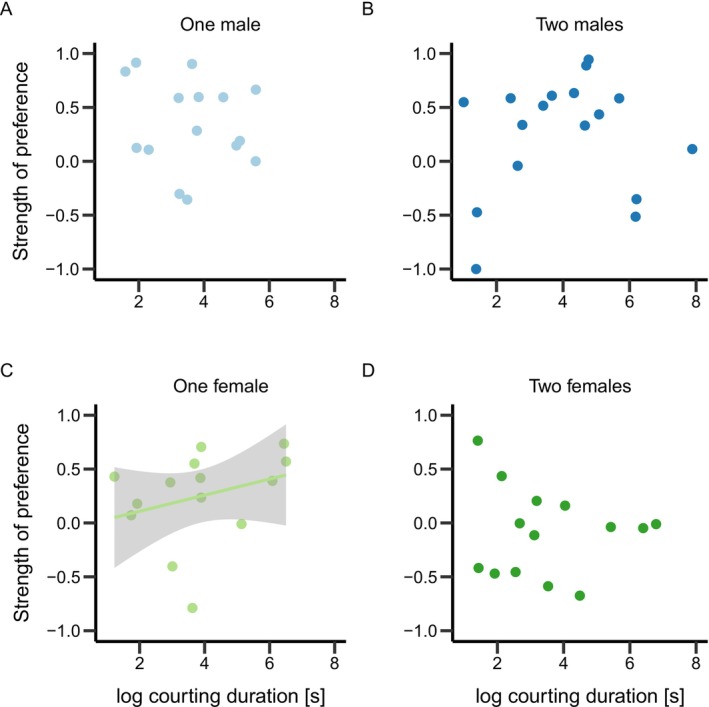
Relationship between strength of preference (SOP) and the log_10_ courting duration of the male in the pair excluding very brief courtship and pairs where the male did not court the female (see Section 2). Data for four different treatment groups (one male (A; *n* = 15), two males (B; *n* = 17), one female (C; *n* = 15) and two females (D; *n* = 14)). Green and blue colors indicate male and female treatment, respectively. Light and dark colors indicate one or two individuals in the treatment group, respectively. Line in panel (C) representing linear model fit with 95% confidence intervals in gray.

**FIGURE A1:**
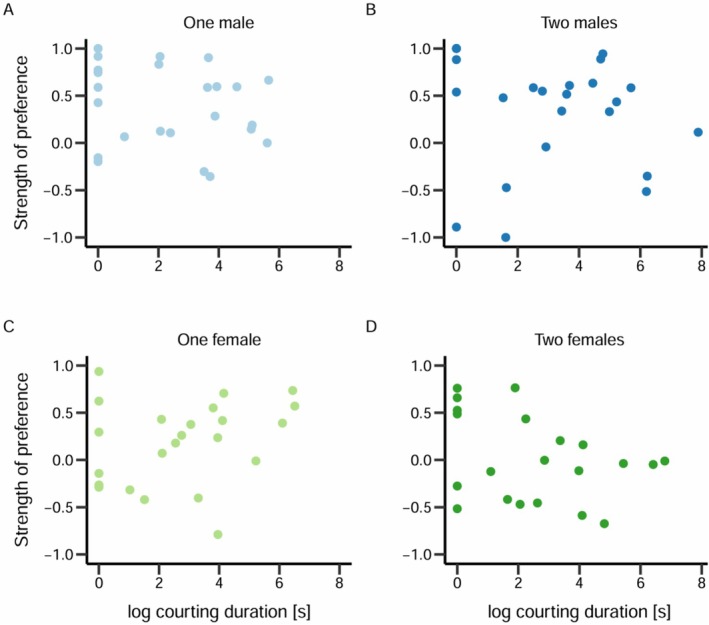
Relationship between strength of preference (SOP) and the log_10_ courting duration of the pair (including pairs where the male did not court) for four different treatment groups (one male (A), two males (B), one female (C) and two females (D)). Green and blue colors indicate male and female treatment, respectively. Light and dark colors indicate one or two individuals in the treatment group, respectively.

Second, we tested if the amount of courtship behavior performed in the pair predicted the association preference of focal males. There were no significant relationships between SOP and courtship behavior (Table A1, Figure A1) when including all courtship data. Similarly, when we performed an analysis excluding pairs where the male did not perform any or only very brief courtship (see Section 2), there were no significant relationships between SOP and courtship behavior in pairs where the male performed courtship in the treatment groups with one male (Spearman correlation, *ρ* = −0.12, df = 14, *p* = 0.676), two males (*ρ* = 0.04, df = 16, *p* = 0.869), or two females (*ρ* = −0.14, df = 13, *p* = 0.627; Figure 4). By contrast, when presented with a pair versus a single other female, the focal males associated more with pairs where the courting male spent more time courting the female (*ρ* = 0.53, df = 14, *p* = 0.045; Figure 4C).

**FIGURE 5:**
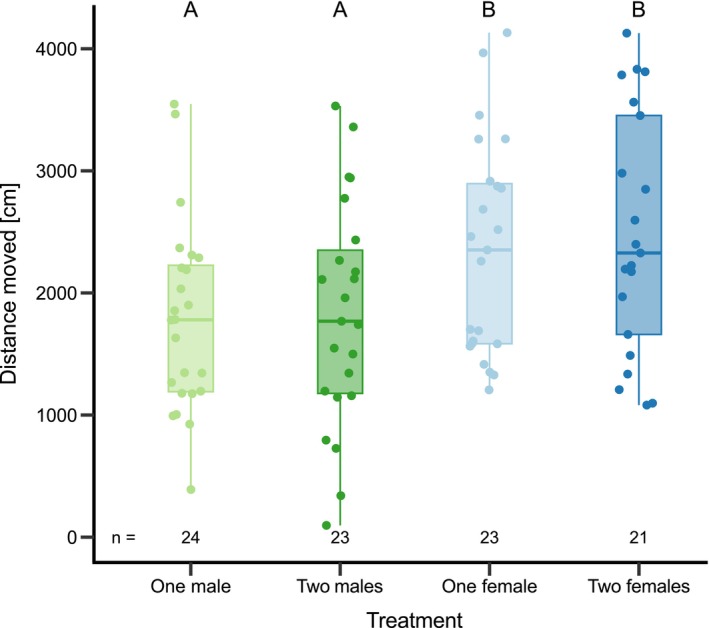
The distance moved by focal males with a choice between a pair and one of four treatment groups (i.e., one male, two males, one female or two females). Green and blue colors indicate male and female treatment, respectively. Light and dark colors indicate one or two individuals in the treatment group, respectively. Different letters denote significant differences between treatments (Table 2b). Boxplots show median, first/third quantiles and whiskers indicating data range excluding outliers.

**FIGURE A2:**
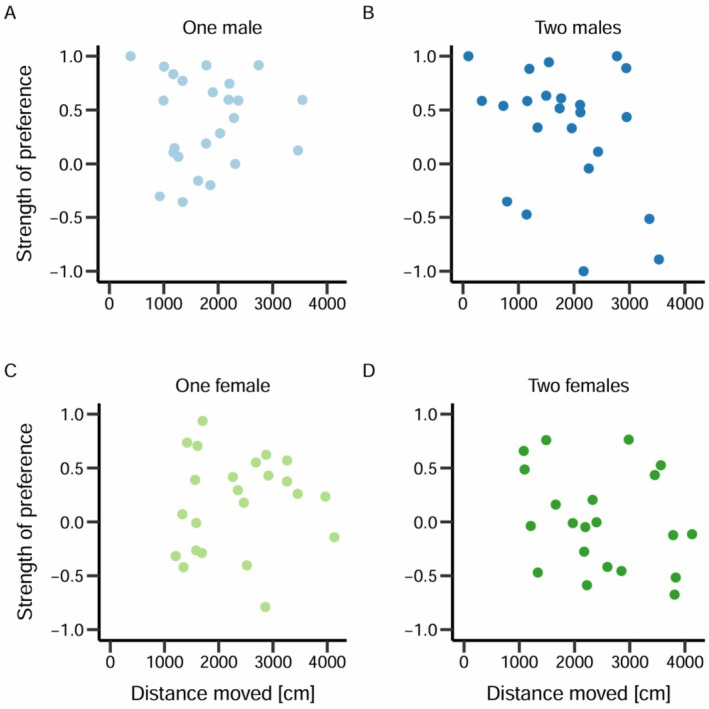
Relationship between strength of preference (SOP) and the distance moved by the focal male for four different treatment groups (one male (A), two males (B), one female (C) and two females (D)). Green and blue colors indicate male and female treatment, respectively. Light and dark colors indicate one or two individuals in the treatment group, respectively.

Third, we explored if general activity patterns (i.e., the total distance moved during the trial) varied with treatment or affected the SOP. The activity of focal males was significantly higher when females were present in the treatment group compared to when males were present (Table 2b, Figure 5). However, the number of fish in the treatment group did not affect the focal male's general activity (Table 2b, Figure 5). Furthermore, there was no relationship between activity and the SOP in either of the treatment groups and there was only a non‐significant negative trend across all treatments (Table A2, Figure A2).

### 3.2 Do Morphological Traits Influence Strength of Preference or Courtship Behaviors?

**FIGURE A3:**
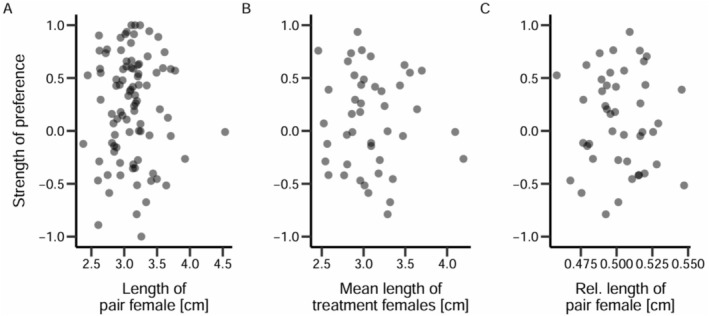
Relationship between strength of preference (SOP) and the length of the pair female (A), the mean length of the treatment females (B), and the length of the pair female relative to the mean length of the treatment females (C).

**FIGURE A4:**
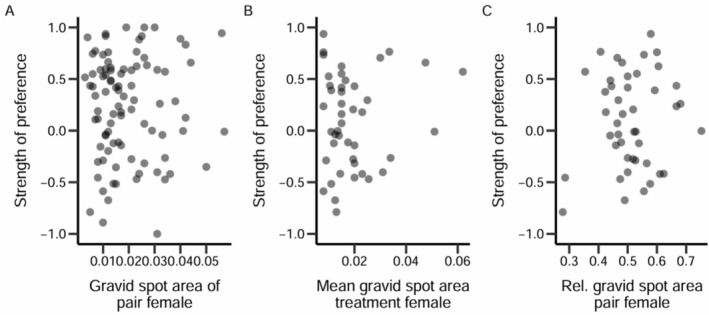
Relationship between strength of preference (SOP) and the gravid spot area of the pair female (A), the mean gravid spot area of the treatment females (B), and the gravid spot area of the pair female relative to the mean gravid spot area of the treatment females (C).

**FIGURE A5:**
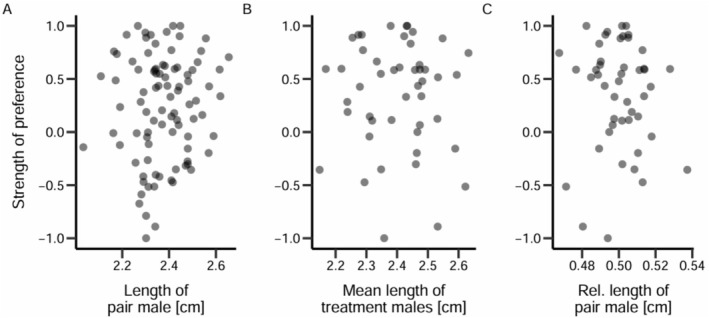
Relationship between strength of preference (SOP) and the length of the pair male (A), the mean length of the treatment males (B), and the length of the pair male relative to the mean length of the treatment males (C).

Next, we tested if the SOP of the focal male was related to any morphological characteristics of the stimulus individuals (both the pair and the treatment individuals). We found no relationships in SOP with body length of both the pair as well as stimulus females nor with the length of the pair female relative to the treatment female(s) (Table A3, Figure A3). Furthermore, we found no relationship between SOP and gravid spot size of the pair and treatment females both in absolute terms and relative to body length (Table A4, Figure A4). In addition, the body length of both the stimulus and the pair males as well as their relative length did not correlate with the focal male's SOP (Table A5, Figure A5).

**FIGURE A6:**
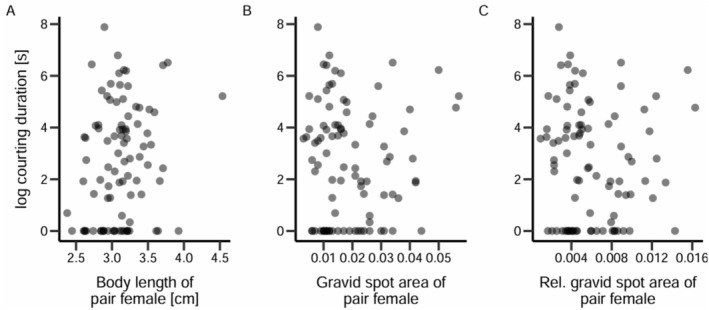
Relationship between pair male courtship duration and the length of the pair female (A), the gravid spot area of the pair female (B), and the gravid spot area of the pair female relative to the mean length of the pair female (C).

**FIGURE A7:**
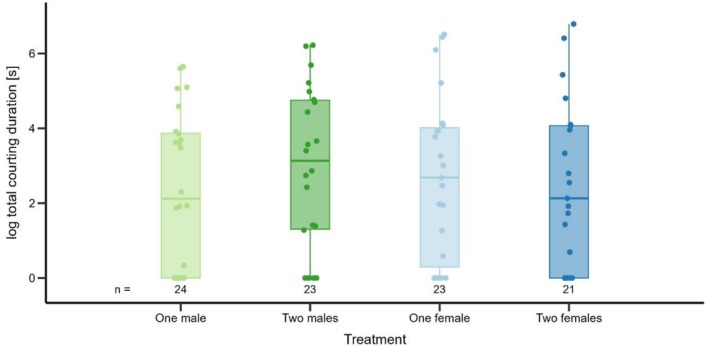
log_10_ courting duration for pair males in assays with four treatment groups (i.e., one male, two males, one female or two females). Green and blue colors indicate male and female treatment, respectively. Light and dark colors indicate one or two individuals in the treatment group, respectively. Sample sizes (*n*) and differences between treatments (Table A7) indicated in figure. Boxplots show median, first/third quantiles and whiskers indicating data range excluding outliers.

Finally, we explored potential factors that influenced the amount of courtship performed by the pair male. We found that both absolute and relative gravid spot size of the pair female did not influence male courtship duration (Table A6, Figure A6). By contrast, males were marginally, non‐significantly more likely to court females that were larger, compared to smaller females (Table A6, Figure A6). Furthermore, the treatment group (i.e., the fish on the other side of the arena) had no significant effect on the courtship of the pair male (Table A7, Figure A7).

## 4 Discussion

We present evidence that males of a shoaling fish strategically associated with conspecifics in four different social scenarios. Specifically, males preferred to associate with a female already paired with another male over other rival males. Males also tended to associate with a female already paired with another male when the alternative option was a single female and adjusted their association behaviors depending on the amount of courtship occurring between the male and female pair. However, males did not show any preference to associate with a female already paired with another male when the alternative option was two females. Contrary to expectations, male halfbeaks did not alter their association preferences based on the number of fish present in either side of the experimental chamber. Overall, our results suggest that male halfbeaks use information about conspecifics, including their sex and potentially courtship activity, to guide association strategies.

We found that halfbeak males preferred to associate with a pair over other males. This behavior could be interpreted either as focal males avoiding rival males or preferring to associate with the female in the pair. Both theoretical (Wedell et al. 2002) and empirical studies (e.g., Schwagmeyer and Parker 1990; Dosen and Montgomerie 2004) suggest that males should adjust their mating behaviors to minimize the risk of sperm competition. In our experiments, if male association strategies were guided by avoiding rival males, we would have expected to find a difference in association between the treatments with one and two males. Specifically, males were predicted to avoid two males more than one, as the potential for conflict should increase with the number of rivals. Yet we found no evidence to support this prediction. Thus, while observing females mating with other males is sufficient to depress male interest in other species (e.g., guppies, *Poecilia reticulata* ; Dosen and Montgomerie 2004), we hypothesize that the clear preference in male halfbeaks to associate with paired females over rival males (either one or two) is driven by the potential mating opportunity presented by the female in the pair. Since halfbeaks habitually live in mixed sex shoals (Devigili et al. 2021), our results suggest that association strategies of males will prioritize potential mating opportunities, even if the female is already paired with another male.

Male halfbeaks' association behaviors when presented with multiple females were more complex. This likely reflects the balance between identifying receptive females and maximizing potential mating opportunities. Focal males were more active in the female compared to the male treatments. This suggests that they spend more effort examining the different stimuli when females were present on both sides. Interestingly, focal males did not associate with a pair of females more than with a female already paired with a male, even though the pair of females provided twice the potential mating opportunities without direct competition. Male halfbeaks also associated more often (albeit non‐significantly) with females paired with a male over a single female. Together, these results suggest that male halfbeaks are not solely focused on avoiding potential sperm competition when making association decisions.

Our results hint at the possibility that male halfbeaks perform mate‐choice copying (Witte et al. 2015), as males showed a tendency to associate with a pair over a single female. Mate‐choice copying is generally expected to be rare in males due to the associated increase in sperm competition risk. However, a recent meta‐analysis suggests that it might be more common than previously thought (Davies et al. 2020). Male mate‐choice copying could evolve in species where sperm competition risk is high throughout the population (Bierbach et al. 2011), female quality or receptivity varies substantially (Ogden et al. 2020), and gathering information about female quality is costly for males (Schlupp and Ryan 1997; Bierbach et al. 2011). All these prerequisites are likely met in the halfbeak fish, as it is a highly polygamous species, with a time‐consuming male courtship behavior that assesses female fertility via the gravid spot (Ogden et al. 2020). Importantly, the courting duration of the pair male predicted the time the focal male spent associating with that pair, but only in pairs where the male was courting and not in pairs where the male did not court at all. Hence, males did not seem to react to an absence of courtship, as a potential indicator of low female quality. Instead, males showed increased association duration toward pairs exhibiting higher courtship intensity. This contradicts patterns observed in other species where males avoid females that are paired with and/or recently mated to a rival male (e.g., Dosen and Montgomerie 2004). Instead, male halfbeaks appear to pay more attention to pairs performing more courtship—but only when the alternative option is a single female. Importantly, we did not find an association of courtship and association preference when two females were presented in the treatment group. Here, the increased potential mating opportunities that two females present might outweigh the benefits of potentially copying the mate‐choice of the paired male. Overall, the relationship between association preference and courtship were weak or absent across our four treatments, which could also indicate a lack of statistical power to detect small effects in a highly plastic trait like mating behavior. Nevertheless, our data suggest that male mate‐choice copying in halfbeaks might only emerge under limited social scenarios (i.e., when choosing between two females where one is courted and one not).

Mate‐choice copying could be an adaptive strategy for male halfbeaks, if there would be second‐male sperm precedence. Unfortunately, sperm precedence patterns of halfbeaks are currently unknown. In addition, it is important to note that the focal male was not able to perform courtship behavior or mate in our setup, as physical contact to the female was prevented by the transparent barrier. Hence, only measured association preferences and not actual mate choice. Nevertheless, association tests are routinely used as a proxy for mate choice in fish (Mautz and Jennions 2011; Callander et al. 2012; Thünken et al. 2012; Booksmythe et al. 2013; Passos et al. 2019; Duffy et al. 2025). While no evidence exists in halfbeaks that association time is correlated with mate choice, studies in other live‐bearing freshwater fishes (e.g., mosquitofish—Vega‐Trejo et al. 2014; guppies—Jeswiet and Godin 2011; and swordtails—Walling et al. 2010) demonstrate that association time can be a reasonable proxy for mate choice in fishes (Dougherty 2020). Ultimately, a test for male mate‐choice copying should not only test association preferences, but should allow free matings and measure sperm allocation across different social scenarios. Future work should aim to directly test for male mate‐choice copying by removing the initially paired male after courtship interactions (Schlupp and Ryan 1997; Bierbach et al. 2011; Nöbel et al. 2018). This approach would help disentangle social influence from individual preference and clarify whether males are actively copying or simply responding to other cues.

Strategically associating with conspecifics is particularly important in shoaling fish, not only because larger groups reduce individual predation risk, but also to find the optimal mating partners (Seghers 1974; A. E. Magurran 1990). We did not find evidence that halfbeaks prefer to associate with the largest possible number of conspecifics, as differences in association were driven by the sex of the treatment individuals and not their number. This matches the findings from Singaporean, but not Malaysian populations of halfbeaks (Ho et al. 2015). In general, fish seem to be able to discriminate between differently sized groups (Xiong et al. 2018). For example, topminnows ( *Girardinus falcatus* ) preferentially associate with larger shoals when presented with two differently sized groups (Agrillo and Dadda 2007). The absence of shoaling effects in the present study could be because of the perceived low predation risk of the individuals in their laboratory environment or due to the small difference in numbers (one vs. two individuals). Previous studies suggest that at low numbers, fish might not choose the larger shoal (Agrillo and Dadda 2007).

We found no effect of gravid spot size on the courtship behavior of the pair male. While a male preference for females with a large gravid spot has previously been shown in pygmy halfbeaks (Ogden et al. 2020), males did not have a choice between different females in the present setting and might have readily courted any female presented to them. Nevertheless, there was a non‐significant tendency for males to court larger females more compared to smaller females. This could be because larger females might have a fecundity advantage over small females, as in other fish species (Cheong et al. 1984; Tsoukali et al. 2016). Furthermore, males did not seem to adjust their courtship behavior to the number of potential competitors on the other side of the tank (i.e., the treatment group). This suggests that direct interactions (or at least closer proximity) between males may be needed to increase the perceived risk of competition.

Association strategies are crucial for social animals living in complex environments (Pavlov and Kasumyan 2000). We situated male halfbeaks in four simplified social scenarios and found that they preferred to associate with a pair over other males and tend to associate with pairs performing courtship behavior, suggesting mate‐choice copying behavior—but only under a limited range of social scenarios. Overall, our simplified social setting illuminates strategic association behaviors in a shoaling fish, but also generates hypotheses (e.g., regarding male mate‐choice copying) to be tested under more natural conditions. Future studies could test how the basic association patterns we observed in a controlled laboratory environment affect shoals of halfbeaks in the wild, potentially integrating population density, sex‐ratio and ecological factors like habitat complexity or predation pressure.

## Author Contributions

**Arezo Shamsgovara:** conceptualization (equal), data curation (lead), investigation (equal), methodology (equal), validation (equal), visualization (supporting), writing – original draft (equal), writing – review and editing (equal). **Lennart Winkler:** data curation (supporting), formal analysis (lead), investigation (equal), methodology (equal), software (lead), supervision (supporting), visualization (lead), writing – original draft (equal), writing – review and editing (equal). **Alvin Sellin:** data curation (supporting), investigation (supporting), writing – review and editing (equal). **David Wheatcroft:** conceptualization (supporting), methodology (supporting), writing – review and editing (equal). **Niclas Kolm:** conceptualization (supporting), methodology (supporting), writing – review and editing (equal). **John L. Fitzpatrick:** conceptualization (lead), funding acquisition (lead), methodology (equal), project administration (lead), resources (lead), supervision (lead), writing – review and editing (equal).

## Funding

This work was supported by the Wenner‐Gren Foundation (UPD2023‐0113) and Vetenskapsrådet (2021‐04615).

## Conflicts of Interest

The authors declare no conflicts of interest.

## Data Availability

All data and code can be accessed through Zenodo (https://doi.org/10.5281/zenodo.16894266; Shamsgovara et al. 2026).

## Appendix A

**TABLE A1:** Spearman correlation coefficients for the relationship between strength of preference (SOP) and the log_10_ courting duration of the pair (including pairs where the male did not court) for four different treatment groups (one male, two males, one female and two females).

Treatment	*ρ*	df	*p*
One male	−0.20	22	0.338
Two males	−0.20	21	0.356
One female	0.26	22	0.236
Two females	−0.20	21	0.356

### Relationship Between SOP and Distance Moved by Treatment

**TABLE A2:** Spearman correlation coefficient (ρ) for the relationship between the distance moved by the focal male and its strength of preference (SOP) for four different treatments (i.e., one male, two males, one female or two females).

Treatment	*ρ*	*p*
One male	0.02	0.917
Two males	−0.31	0.145
One female	0.15	0.495
Two females	−0.32	0.156
Across all treatments	−0.20	0.062

### Relationship Between Stimulus Morphology on SOP

#### Body Length of Females

**TABLE A3:** Spearman correlation coefficient (*ρ*) for the relationship between focal male strength of preference (SOP) and the length of the pair female, the mean length of the treatment females and the length of the pair female relative to the mean length of the treatment females.

Trait	*ρ*	*p*
Lenth of pair female	−0.01	0.937
Mean length of treatment females	−0.04	0.795
Relative length of pair female	−0.08	0.590

#### Gravid Spot Area

**TABLE A4:** Spearman correlation coefficient (*ρ*) for the relationship between focal male strength of preference (SOP) and the gravid spot area of the pair female, the mean gravid spot area of the treatment females and the gravid spot area of the pair female relative to the mean gravid spot area of the treatment females.

Trait	*ρ*	*p*
Gravid spot area of pair female	0.01	0.887
Mean gravid spot area of treatment females	−0.01	0.941
Relative gravid spot area of pair female	0.02	0.890

#### Male Body Length

**TABLE A5:** Spearman correlation coefficient (*ρ*) for the relationship between focal male strength of preference (SOP) and the length of the pair male, the mean length of the treatment males, and the length of the pair male relative to the mean length of the treatment males.

Trait	*ρ*	*p*
Lenth of pair male	0.08	0.469
Mean length of treatment males	−0.09	0.552
Relative length of pair male	−0.11	0.453

### Relationship Between Male Courtship Behavior and Female Morphology

**TABLE A6:** Spearman correlation coefficient (*ρ*) of the relationship between pair male courtship duration and the length of the pair female, the gravid spot area of the pair female, and the gravid spot area of the pair female relative to the length of the pair female.

Trait	*ρ*	*p*
Lenth of pair female	0.19	0.074
Gravid spot area of pair female	−0.04	0.723
Relative gravid spot area of pair female	−0.08	0.459

### Effect of Treatment on Courting Duration

**TABLE A7:** Comparisons of log_10_ courting duration between the treatment sex (i.e., male or female) and the number of fish in the treatment group (i.e., one or two), as well as post hoc comparisons between all treatment groups.

Treatment	Estimate	SE	*t* value	*p*
Sex (male)	0.25	0.47	0.53	0.595
Number of fish (two)	0.33	0.47	0.70	0.484
One male—two males	0.68	0.66	1.04	0.302
One female—two females	−0.04	0.68	−0.06	0.949
One male—one female	0.10	0.68	0.15	0.880
One male—two females	0.05	0.67	0.08	0.935
Two males—one female	−0.58	0.66	−0.88	0.382
Two males—two females	−0.63	0.68	−0.92	0.359

*Note:* dfs were 88 and 87, respectively. Reference group given in brackets.
